# Assessment of behavioral, neuroinflammatory, and histological responses in a model of rat repetitive mild fluid percussion injury at 2 weeks post-injury

**DOI:** 10.3389/fneur.2022.945735

**Published:** 2022-10-20

**Authors:** Katherine M. Fronczak, Andrea Roberts, Sarah Svirsky, Madison Parry, Erik Holets, Jeremy Henchir, C. Edward Dixon, Shaun W. Carlson

**Affiliations:** ^1^Neurological Surgery, University of Pittsburgh, Pittsburgh, PA, United States; ^2^VA Pittsburgh Healthcare System, Pittsburgh, PA, United States

**Keywords:** traumatic brain injury, behavioral impairment, histopathology, neuroinflammation, model characterization

## Abstract

Repetitive mild traumatic brain injury (rmTBI) is a prominent public health concern, with linkage to debilitating chronic sequelae. Developing reliable and well-characterized preclinical models of rmTBI is imperative in the investigation of the underlying pathophysiological mechanisms, as models can have varying parameters, affecting the overall pathology of the resulting injury. The lateral fluid percussion injury (FPI) model is a reliable and frequently used method of TBI replication in rodent subjects, though it is currently relatively underutilized in rmTBI research. In this study, we have performed a novel description of a variation of the lateral repetitive mild FPI (rmFPI) model, showing the graded acute behavioral impairment and histopathology occurring in response to one, two or four mild FPI (1.25 atm) or sham surgeries, implemented 24h apart. Beam walking performance revealed significant motor impairment in injured animals, with dysfunction increasing with additional injury. Based upon behavioral responses and histological observations, we further investigated the subacute pathophysiological outcomes of the dual FPI (dFPI). Immunoreactivity assessments showed that dFPI led to regionally-specific reductions in the post-synaptic protein neurogranin and increased subcortical white matter staining of the presynaptic protein synaptophysin at 2 weeks following dFPI. Immunohistochemical assessments of the microglial marker Iba-1 showed a striking increase in in several brain regions, and assessment of the astrocytic marker GFAP showed significantly increased immunoreactivity in the subcortical white matter and thalamus. With this study, we have provided a novel account of the subacute post injury outcomes occurring in response to a rmFPI utilizing these injury and frequency parameters, and thereby also demonstrating the reliability of the lateral FPI model in rmTBI replication.

## Introduction

Traumatic brain injury (TBI) is a leading public health concern worldwide, with mild injuries accounting for 80–90% of all TBIs (1–4). Mild TBI (mTBI), which has become synonymous with concussion, has received rapidly growing interest in the research community due to its high prevalence in athlete and military populations, the incidence of long-term consequences, and its link to neurodegenerative pathologies (5–8). Clinically, a mTBI is characterized by a lack of anatomical abnormalities, as recognized by a computerized tomography scan or magnetic resonance imaging (2, 9). Though mortality and functional dependence are not typically observed following mild brain trauma, incidences of repeated mild TBI (rmTBI) have been linked to a battery of debilitating chronic sequelae including depression, irritability, learning and memory impairment, an increased risk of chronic traumatic encephalopathy (CTE), and other long-term symptoms (2, 6, 9–15). Clinical and preclinical studies have shown that repeat concussive events can be compounding in terms of susceptibility and symptomology, however the underlying pathophysiological mechanisms of rmTBI remain an area of intense study (6, 9, 11, 12, 16, 17).

Presently, findings from preclinical studies reveal that mTBI is routinely associated with neurobehavioral dysfunction, indicators of histopathology, and neuroinflammatory responses. Neurobehavioral measures remain fairly consistent across varying rat and mouse preclinical models of rmTBI, in that repeated mild injury tends to produce some degree of motor deficit, although reports of significant learning and memory impairment are less consistent (18–25). In terms of histopathological changes, repetitive mild brain injury is not associated with gross morphological damage or overt neuronal loss, but instead is linked with more subtle microstructural damage such as axonal abnormalities (20, 26–31). Studies detailing neuroinflammation have observed robust activation of astrocytes and microglia following repeated mild injury in multiple experimental models (18, 22, 25, 29, 31–34).

Preclinical models of rmTBI have demonstrated that the severity of pathological outcomes following injury are highly dependent upon the number of injuries received and rest time between impacts (inter-injury interval), supporting the idea of a period of increased vulnerability following additive mTBIs, previously described as the compounding effect (18, 20, 23, 27, 29, 35–38). Due to the heterogeneity of human concussion, there has been a call for investigators to produce comprehensive and thorough accounts of rmTBI models in terms of injury implementation and pathophysiological response in order to confidently compare outcomes across studies and clarify differing response profiles (16, 17).

With this study, we sought to describe the pathological outcomes of a specific variation of rmTBI, using an accessible and underutilized injury application of lateral fluid percussion injury. We have investigated the compounding influence of lateral repeated mild fluid percussion injuries (rmFPI) on acute neurobehavioral responses in the 2 weeks post-injury in animals receiving one, two, and four mild FPI, occurring 24 h apart. Qualitative analysis of Cresyl violet staining revealed that overt tissue damage is present following four mild FPI, with reduced evidence of tissue damage following one mild FPI and two mild FPI. In response to these observations, we sought to further investigate the neuroinflammatory responses and synaptic protein changes following the dual FPI (dFPI) at 2 weeks post-injury, to assess subacute outcomes at the onset of increased vulnerability. Microglial response was assessed by measuring ionized calcium-binding adaptor molecule 1 (Iba-1) immunoreactivity, astrocytic response by assessing glial fibrillary acidic protein (GFAP), and synaptic changes were evaluated by measuring the presynaptic synaptophysin and post-synaptic neurogranin immunoreactivity. This study not only provides a novel account of subacute post-injury outcomes associated with this specific variation of rmTBI, but also serves to demonstrate the efficacy and reliability of lateral FPI in rmTBI studies.

## Materials and methods

### Animals

All experimental procedures were approved by the University of Pittsburgh Institutional Animal Care and Use Committee in accordance with the guidelines established by the National Institutes of Health in the Guide for the Care and Use of Laboratory Animals. Adult male Sprague Dawley rats (Envigo, Indianapolis, IN), aged 8–12 weeks, were housed up to two rats per cage in the University of Pittsburgh vivarium with a 12:12 light/dark photoperiod (lights on at 7:00 am) and with *ad libitum* access to food and water. A total of 60 rats were utilized in the current study, and the breakdown of the groups is detailed below for the multiple outcomes assessed.

### Repeated mild lateral fluid percussion injury

This study consists of two cohorts of animals with *n* = 12 rats per injury group in each cohort. Cohort 1 (*n* = 36) contains animals subjected to a single lateral FPI (sFPI), four lateral FPI (qFPI), or four sham control surgeries (qSh), and cohort 2 (*n* = 24) contains animals subjected to two lateral FPI (dFPI) or two sham surgeries (dSh). In an effort to reduce animal numbers in cohort 1, the qSh was selected in order to control for maximal number of surgical incidences and anesthesia exposures, and thus a single sham control group was not included. Rats were anesthetized using 4% isoflurane in a 2:1 N_2_O/O_2_ mixture within a ventilated anesthesia chamber. Following endotracheal intubation, rats were mechanically ventilated, and anesthesia maintained with 2% isoflurane in a 2:1 N_2_O/O_2_ mixture. Animals were placed in a stereotaxic frame in the prone position, body temperature was monitored by rectal thermistor probe and maintained at 37°C using a heating pad. Following a midline incision, the soft tissues were reflected. A craniectomy was completed, using a 4.7 mm trephine, between bregma and lambda, centered 5 mm lateral of the sagittal suture to expose the dura mater over the right parietal cortex. Set screws were secured in pilot holes at least 5 mm from the craniotomy. A Luer-lock hub was fitted to the craniotomy and secured with cyanoacrylate gel to adhere and seal the hub to the skull. The hub was secured to the set screws by application of methyl-methacrylate (Henry Schein, Melville, NY, USA). Once the methyl methacrylate was hardened, the hub was filled with sterile saline and connected to the injury device. All animals were randomly assigned to the injury group in the first surgical procedure. Sham animals were subjected to all surgical procedures except the induction of the injury. For induction of the FPI, the pendulum hammer was released onto the piston of the fluid filled cylinder to induce the injury (1.25 atm; AmScien Instruments, Richmond, VA). Rats receiving multiple sham surgeries or FPI were re-anesthetized and received subsequent control or fluid percussion injuries 24 h after each preceding injury (Table 1). For repeated procedures, the hub remained attached for all repeated surgical procedures and between procedures, the hub was filled with sterile artificial cerebrospinal fluid, topped with a sterile rubber cap and the head sutured. Following completion of the final surgical procedure, the hub was removed, and the head sutured. Health indicators of body weight, apnea time and righting reflex time were recorded for each animal immediately following injury. Once ambulatory, the animals were returned to their home cage. Anesthesia times were considerably longer on injury day 1 reflective of the initial surgical procedures completed including the craniotomy, set screws and installation of the injury hub. Beginning at the completion of the first surgical procedure, all animals were provided *ad libitum* standard food chow mush (milled food chow + water) in the cage daily for up to 5 days following the last procedure.

**Table 1:** Injury schedule.

**Injury group**	**D1**	**D2**	**D3**	**D4**
**(A) Cohort 1**				
qSh	Sham	Sham	Sham	Sham
sFPI	mFPI	-	-	-
qFPI	mFPI	mFPI	mFPI	mFPI
**(B) Cohort 2**				
dSh	Sham	Sham	-	-
dFPI	mFPI	mFPI	-	-

Representation of mFPI (mild fluid percussion injury) and sham control surgery schedule, day 1–4 (D1-D4). (A) Schedule for cohort 1, including qSh (quad sham), sFPI (single fluid percussion injury), and qFPI (quad fluid percussion injury) groups. (B) Schedule for cohort 2, including dSh (dual sham) and dFPI (dual fluid percussion injury).

### Vestibular motor function

All behavioral tasks were completed separately for each cohort. A total of 60 rats (n = 12 for each injury status) were utilized to evaluate vestibular motor function and spatial learning and memory similar to previously described (39–41). Assessments of beam balance and beam walking were completed on days 1–5 post-injury with training completed on day 0 immediately prior to injury. Vestibulomotor function was evaluated with a beam balance task. The animal was placed on a suspended, narrow wooden beam (1.5 cm width) 30″ above a padded surface. The latency on the beam, maximum of 60 s, was measured. Each animal was tested over three trials per day with a 30 s rest period between trials. A modified beam walking task was utilized to evaluate fine motor components of vestibular motor function. Prior to injury, animals were trained to escape a loud pink noise by traversing a narrow wooden bean (2.5 x 100 cm) to enter a darkened goal box at the end of the beam. Four pegs (4 cm high and 3 mm in diameter) were equally spaced across the length of the beam to increase the difficulty of the test. If the rats fell off the beam or did not traverse the beam in the allotted 60 s period, the animal was placed back on the beam and guided to the goal box; the noise was turned off once the animal reached the box. Performance was assessed by recording the average latency to traverse the beam.

### Spatial learning and memory

Spatial learning and memory were evaluated in the same rats subjected to vestibular motor testing, again with cohorts assessed separately, using the Morris water maze (MWM) task via a video-tracking system (AnyMaze, Stoelting, Inc., Wood Dale, IL, USA) as previously described (39, 41, 42). A 180 cm circular tank (45 cm high) was filled with 21 ± 1°C water to a height of 30 cm to conceal a transparent circular platform (10 cm in diameter and 29 cm high) in a fixed location positioned 45 cm from the wall. Visual cues located on the walls outside the tank aid in locating the escape platform. Testing began on day 9 post-injury, without previous exposure or training to the MWM task, and continued for 5 days (days 9–13 post-injury) with each animal completing four trials per day. Rats were randomly placed in the water against the wall and released to swim in the tank to find the hidden platform in a 120 s period. In the event the animal was unable to locate the platform within the allowed time, it was manually directed to the platform. The rat remained on the platform for 30 s and was placed in an incubator between trials. Following a 4 min inter-trial interval, the subsequent trial was initiated. On day 14 post-injury, the animal was tested using a probe trial paradigm in which the hidden platform was removed. Probe trial times were utilized to assess probe trial performance between the group.

### Tissue preparation for histopathological analysis and serum measurements by ELISA

A total of *n* = 30 rats (*n* = 6 per group in randomly selected from each cohort) were sacrificed at 14 days following the final FPI or sham control surgery. Animals received an overdose of Fatal-plus (intraperitoneally, 100 mg/kg, sodium pentobarbital (Fatal-Plus), Vortech Pharmaceuticals, Dearborn, MI), whole blood was transcardially collected, and then the animal was transcardially perfused with USP saline, followed by 10% neutral buffered formalin (Fischer Scientific, Waltham, MA). The brains were post-fixed in 10% neutral buffered formalin for an additional 24 h, cryoprotected with 30% sucrose in 0.1 M phosphate buffered saline (PBS) for 72 h at 4°C, and frozen in Tissue-Tek OCT compound (Sakura Finetek, Torrance, CA). Brains were cut into 35 μm thick coronal sections using a cryostat (Leica Microsystems Inc., Buffalo Grove, IL) and collected for histological and immunohistochemical outcomes. Whole blood was allowed to clot for 30 min at room temperature, centrifuged at 1,500 x g, and supernatant collected and stored at −80°C prior to use. ELISA measurements for IL-6 (R&D Systems, Cat #R6000B) and CCL2 (R&D Systems, Cat#DY3144) were completed for measurement of the levels of each analyte in serum in accordance with the manufacturer's instructions.

### Qualitative analysis of histopathology and neurodegeneration

Sections from each animal, ranging from −6.5 to 4.2 mm bregma, spaced 500 μm apart, were selected and mounted on gelatin-subbed slides, cleared in xylene, and hydrated in 100, 95, and 70% ethanol, followed by distilled water before incubation in Cresyl violet stain. The slides were then rinsed in distilled water before being dehydrated in ethanol, cleared in xylene, then cover slipped using Permount medium (Fischer Scientific). Large image scans of stained brain slices were acquired at 10x magnification and merged into one image incorporating both hemispheres using a C2 Nikon 90i microscope. Histopathology was assessed between the groups by visually comparing overt tissue changes, including evidence of cortical thinning, and overt alterations in the hippocampus, thalamus, and subcortical white matter. The neurodegenerative marker Fluoro-Jade B (FLB) was utilized to label degenerating neurons at 2 weeks following dFPI. Staining was completed as previous described (43). Fluoro-Jade C labeled neurons were imaged with a 4X objective using the Nikon 90i microscope.

### Immunohistochemistry

Based on post-injury measures, behavioral outcomes, and histopathological outcomes, animals receiving dSh and dFPI (n=6 per group) were selected to be assessed for immunoreactivity changes. From the same animals utilized for histopathology analysis, sections ranging from −3.2 mm to −4.20 mm bregma were selected for immunohistochemical staining as these bregma sections encompass the primary site of injury. Immunohistochemical staining was completed in 24 well-plates on free-floating tissue sections, using one section from each animal for each protein. The sections were rinsed with 0.1 M tris-buffered saline (TBS) buffer and blocked with 10% normal goat serum and 0.1% Triton X-100 in 0.1M TBS (TBS-T) for 1 h. Sections were incubated overnight at 4°C with anti-ionized calcium binding adapter molecule 1(Iba1) antibody (Rabbit, 1:2,000, Fujifilm Wako, Cat# 019-19741), anti-GFAP (Rabbit, 1:5,000, Millipore, Cat# AB5804), anti-synaptophysin antibody (Rabbit, 1:2000, Abcam, Cat# 32127) or Rabbit anti-neurogranin (Rabbit, 1:10,000, Abcam, Cat# 217672). The following day, sections were rinsed with TBS-T, incubated with secondary antibody, followed by horseradish peroxidase, and rinsed with TBS-T to prepare for substrate development. For each protein, all sham control and FPI tissues were run simultaneously. Diaminobenzidine (DAB) was added, and the reaction development time was controlled to ensure equal DAB substrate exposure time between all sections within each protein (SK-4100, Vector Laboratories, Burlingame, CA). Sections were mounted on Superfrost Plus slides (Fischer Scientific) and were cover-slipped using Permount medium.

### Assessment of immunohistochemistry

To quantify pixel intensity differences in immunohistochemical staining of dSh and dFPI brains, pixel intensity was assessed, as previously described by Fronczak et al. (39). Large image scans of stained brain slices were acquired at 10x magnification and merged into one image incorporating the cortex, hippocampus, and thalamus using a C2 Nikon 90i microscope. Images for Iba1 and synaptophysin were converted to 32-bit grayscale and inverted to prepare for mean pixel intensity measurements using ImageJ. The hippocampus, cortex, subcortical white matter, and thalamus for each section were identified, with regional boundaries defined by the rat brain atlas (44). The polygon tool was used to delineate the described regions of the ipsilateral brain to complete measurements of mean pixel intensity for each region. The mean pixel intensity for the medial corpus callosum was subtracted from the mean pixel intensity of each region in the same section to normalize background staining intensity for every section. Mean pixel intensity measures of each region on the ipsilateral hemisphere were averaged. For each region, an average group mean of normalized pixel intensities were calculated and normalized as a percentage of ipsilateral sham intensity. 20x magnification large image scans were also acquired for subregions and displayed as image inserts to show specific staining phenomena.

### Statistical analysis

All quantification was completed by an investigator blinded to the injury status of each animal. Data are presented as mean ± standard error of the mean (SEM). Weight, apnea time, and righting time were recorded immediately following each injury or sham surgery. Weights, apnea times, and righting times measured on injury days 1 and 2 were compared on each day with a repeated measures one-way ANOVA. When appropriate, a Tukey's *post hoc* multiple comparisons test was completed. Weights and righting times following qSh or qFPI measured on days 3 and 4 were compared on each day using a student's *t*-test. Beam walking latency and Morris water maze performance for sham groups from both cohorts (qSh and dSh) were compared using a repeated measures one-way ANOVA. Statistical analyses for the two cohorts demonstrated that the shams from each cohort were not significantly different in terms of neurobehavioral performance and were determined to be statistically capable of being combined. Subsequent to this, statistical comparisons of beam balance latency, beam walking latency and spatial acquisition were completed together with both cohorts and combined sham groups using a repeated measures one-way ANOVA, followed by Tukey's multiple comparisons test when appropriate. Probe trial percent time was compared using a one-way ANOVA. Serum measurements of CCL2 by ELISA were compared by Student's t-test. Mean pixel intensity measurements of immunohistochemical staining for dFPI animals were analyzed by Student's t-tests assessing the difference between dSh and dFPI groups for each region. Statistical tests were completed using GraphPad (GraphPad version 9, La Jolla, CA). A *p*-value < 0.05 was considered statistically significant for all tests.

## Results

The study design and implementation of repeated injuries is highlighted in Table 1. The average apnea and righting times following every sham control or mild FPI are shown in Table 2, along with the average weights, measured at the time of induction of either sham or FPI injures. There were no significant differences in weights between groups at the time of injury compared on days 1 and 2. However, there was a significant difference between weights of qSh and qFPI animals on days 3 and 4 (Student's *t*-tests; *p* < 0.05; Table 2). There were no significant differences in apnea times of sFPI, dFPI, and qFPI groups on injury day 1, and also no differences between dFPI and qFPI groups on injury day (Table 2). There were significant differences in righting times in all injured groups compared to sham groups on injury days 1 and 2 (ANOVA; *p* < 0.0001; Table 2), but there were no significant differences between injury groups. There were significant differences in righting times between qSh and qFPI groups on days 3 and 4 (Student's *t*-tests; *p* < 0.0005; Table 2). These post-injury measures suggest that injury severity is comparable across groups and that there were no significant differences between the sham groups of the two cohorts.

**Table 2:** Post-injury measures.

**Injury group**	**Weight 1 (g)**	**Weight 2 (g)**	**Weight 3 (g)**	**Weight 4 (g)**
**(A) Weight (g)**				
qSh	320.3 ± 6.2	316 ± 5.6	318.9 ± 5.7	316.2 ± 5.6
sFPI	330.3 ± 5.9	-	-	-
qFPI	322.5 ± 5.6	310 ± 6.9	302.9 ± 6.1^*^	294.4 ± 6.1^*^
dSh	321.6 ± 3.8	311.6 ± 3.5	-	-
dFPI	322.4 ± 5.8	307.9 ± 2.9	-	-
**(B) Apnea time (s)**				
**Injury group**	**Apnea 1 (s)**	**Apnea 2 (s)**	**Apnea 3 (s)**	**Apnea 4 (s)**
qSh	-	-	-	-
sFPI	24.8 ± 2.4	-	-	-
qFPI	23.4 ± 4.4	29.5 ± 6.4	15.3 ± 1.6	10.9 ± 1.3
dSh	-	-	-	-
dFPI	12.5 ± 1.2	29.1 ± 8.9	-	-
**(C) Righting time (s)**				
**Injury group**	**Righting 1 (s)**	**Righting 2 (s)**	**Righting 3 (s)**	**Righting 4 (s)**
qSh	154.1 ± 16.6	106.3 ± 12.3	125.3 ± 14.9	84.1 ± 13.3
sFPI	529.3 ± 62.4^*^	-	-	-
qFPI	405.4 ± 49.1^*^	343.6 ± 32.7^*^	261.0 ± 30.4^*^	334.8 ± 24.8^*^
dSh	128.5 ± 23.8	132.4 ± 12.9	-	-
dFPI	356.4 ± 15.1^*^	392.9 ± 24.2^*^	-	-

Representation of acute post-injury outcomes (mean ± SEM) following each injury or sham control surgery for each group on each day. (A) There were no significant differences in weights measured between any of the groups compared on injury days 1 and 2. There were significant differences in weights of qSh and qFPI animals on days 3 and 4 (Student's t-test,

* p < 0.05). (B) Injured groups did not differ in measures of apnea compared to each other in injury day 1 and 2. (C) There were significant differences in righting times in injured groups compared to sham groups injury days 1 and 2 (repeated measures one-way ANOVA, p < 0.0001), while there were no differences between dSh and qSh groups on these days. There were significant differences in righting times in qSh and qFPI animals on injury day 3 and 4 (Student's t-test, p < 0.0005).

Neurobehavioral testing was completed to determine how behavioral functioning is affected by rmFPI. Motor and learning performance for the sham groups of the two cohorts (qSh and dSh) were compared at first to determine if outcomes could be compared across cohorts. Statistical analysis showed no significant difference in beam walking (repeated measures one-way ANOVA; main group effect p = 0.429, *F* (1.22) = 1.541) or spatial acquisition performance (repeated measures one-way ANOVA; main group effect *p* = 0.216, *F* (1.22) = 1.619). Based upon these results and the consistency of the post-injury measures, sham animals were combined into one group (*n* = 24) for the remainder of the behavioral analysis, and all injury groups were compared regardless of cohort.

**Figure 1:**
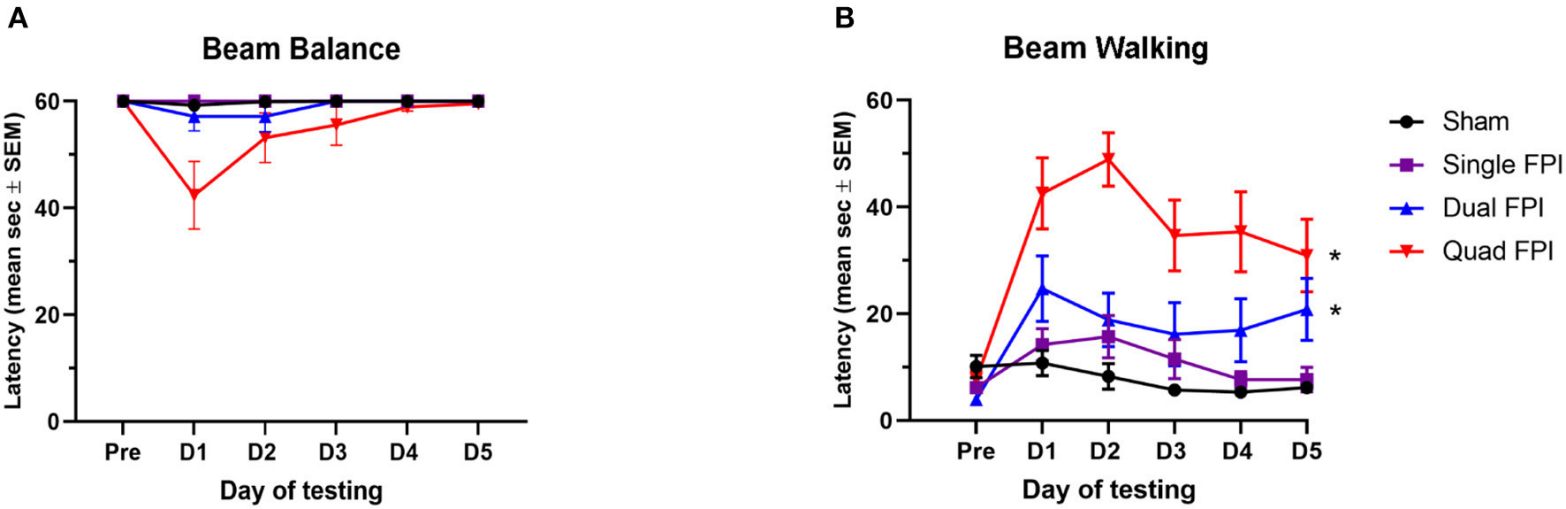
RmFPI results in compounding motor impairment over 5 days post-injury. Analysis performed with sham groups (dSh and qSh) combined. **(A)** Assessment of beam balance latency showed no significant differences in latencies between the groups over the 5 day testing period (repeated measures one-way ANOVA; *p* = 0.2184). **(B)** Assessment of acute vestibular motor function in the beam walking task revealed significant differences in performance between groups over the testing period (repeated measures one-way ANOVA; main group effect *p* < 0.0005). Performances of dFPI and qFPI animals were significantly different than the combined shams (*post-hoc* test, *p < 0.001). All injured groups (sFPI, dFPI, qFPI) were significantly different from each other (*post-hoc* test, *p* < 0.05).

Testing of acute vestibular motor function was completed in the first 5 days following the final FPI to determine how motor functioning is affected by rmFPI, and how the degree of impairment is affected by number of injuries. The beam balance task revealed no significant differences in balance latencies over the 5 day testing period (repeated measures one-way ANOVA; *p* = 0.2184; Figure 1A). The beam walking task revealed significant differences in the latencies over the testing period between the groups (repeated measures one-way ANOVA; main group effect ^*^p < 0.0005, *F* (1.245, 4.979) = 88.83; Figure 1B). Latencies of dFPI and qFPI were significantly different from the combined sham group (*post-hoc* test, ^*^p < 0.001; Figure 1B). All injured groups (sFPI, dFPI, and qFPI) were significantly different from each other over the 5 day testing period (*post-hoc* test, *p* < 0.05).

**Figure 2:**
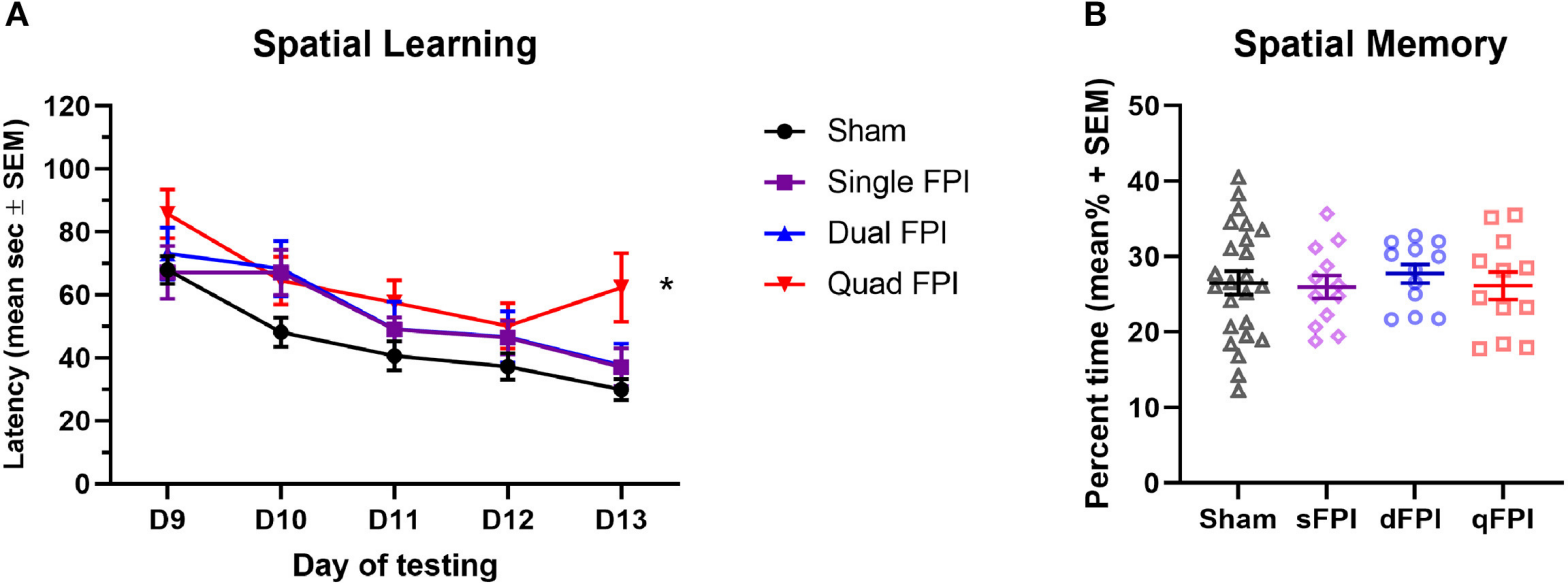
Quad FPI results in impaired spatial learning performance on days 9–13 post-injury. Analysis performed with sham groups (dSh and qSh) combined. **(A)** Assessment of spatial learning on days 9–13 post-injury revealed significantly different performances between groups (repeated measures one-way ANOVA; main group effect *p* < 0.05). Learning latencies showed a significant increase in qFPI compared to combined shams over the 5 day testing period (*post-hoc* test, *p < 0.05). dFPI showed a modest trend in increased latencies compared to the sham group (*post-hoc* test, *p* = 0.0565). **(B)** Assessment of spatial memory using the hidden probe trail on day 14 revealed no significant differences in latencies between the groups.

Spatial learning and memory performance was assessed using the MWM task on days 9–13 following the final injury. Spatial learning latencies revealed significantly different performances between the groups (repeated measures one-way ANOVA; main group effect *p* < 0.05, *F* (1.414, 5.654) = 9.681; Figure 2A). Learning latencies were significantly different between the qFPI and combined sham group (*post-hoc* test, *p* < 0.05; Figure 2A), with a modest trend between dFPI and combined sham groups (*post-hoc* test, *p* = 0.0565; Figure 2A). In the hidden platform probe trial on day 14 post-injury, there were no significant differences in time to locate the platform between groups (Figure 2B).

**Figure 3:**
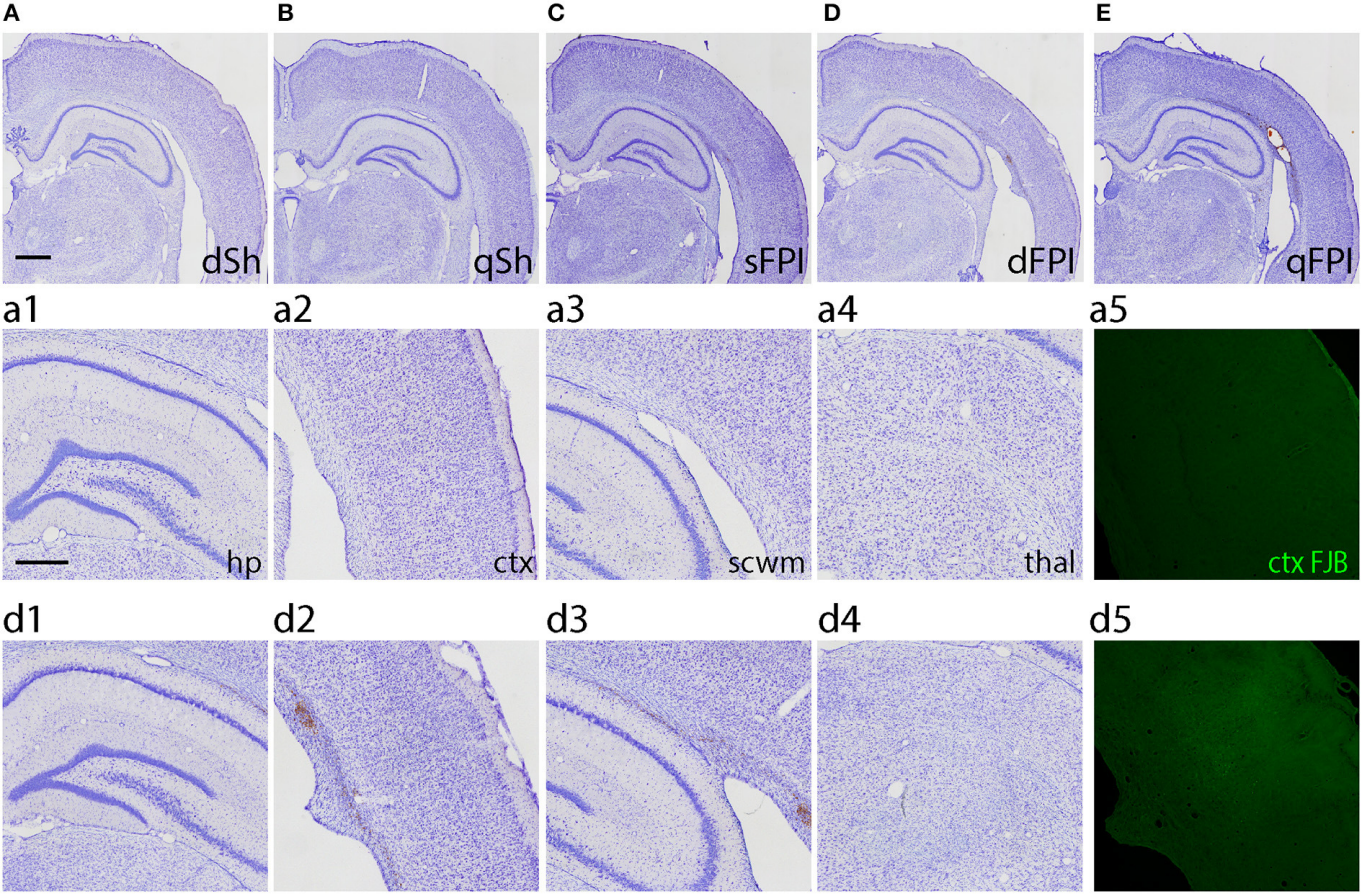
Increasing frequency of injury produces histopathology 2 weeks post-injury. Representative images of dSh **(A)**, qSh **(B)**, sFPI **(C)**, dFPI **(D)**, and qFPI **(E)** Cresyl violet stained sections are shown (total of *n* = 6 per group), scale bar equal to 1 mm. QFPI led to visible tissue damage, including hemorrhage at the interface of the gray matter and white matter, as compared to shams, sFPI, and dFPI. Representative higher magnification images of those shown in dSh **(B)** and dFPI **(D)** show the ipsilateral hippocampus, cortex, subcortical white matter, and thalamus of the Cresyl violet-stained dSh **(a1–a4)** and dFPI **(d1–d4)**, scale bar equal to 500 μm **(d3)**. DFPI led to minimal tissue damage compared to dSh, however it did lead to mild hemorrhaging in the subcortical white matter. Staining with the neurodegenerative marker Fluoro-Jade B revealed positive neurons in the cortex following dFPI, but not following dFPI **(a5,d5)**.

Histopathology was qualitatively assessed by Cresyl violet staining for all injury groups to determine if the injury type led to visible tissue damage. Representative images of Cresyl violet staining for dSh, qSh, sFPI, dFPI, and qFPI are shown (Figures 3A–E, respectively). Representative images of subregions (hippocampus, cortex, subcortical white matter, thalamus) collected from dSh (Figures 3a1–a4) and dFPI (Figures 3d1–d4) are also shown. Qualitative analysis of Cresyl violet staining revealed tissue damage, including potential cortical thinning, visible as a depression in the convexity of the cortex (Figure 3E), and tissue loss at the interface of the gray and white matter tracts following qFPI as compared to shams, sFPI and dFPI. The incidence of this tissue damage was observed in all animals of the qFPI group (6 out of 6). Qualitative analysis of Cresyl violet staining revealed evidence of hemorrhaging and regions of tissue loss at the interface between the gray and white matter following four mild FPI. However, while the incidence of hemorrhage in the dFPI group (6 out of 6) was similar to qFPI, the extent of hemorrhage within the white matter and magnitude of damage appeared to be reduced with fewer injuries, as well as a further reduction in incidence in the sFPI animals (3 out of 6). In combination with behavioral outcomes, the observation of reduced visible tissue damage of dFPI (Figure 3D), as compared to qFPI (Figure 3E), supported further investigation of the pathological outcomes of the dFPI. Qualitive analysis of subregional images showed areas of potential tissue alterations in dFPI as compared to dSh (Figures 3A,D). Assessment of the subcortical white matter after dFPI suggested evidence of hemorrhage in the subcortical white matter, and in the interface between the gray matter and white matter (Figures 3d2,d3). This evidence of hemorrhage was observed in all animals in the dFPI cohort (6 out of 6). To assess for degenerating neurons after dFPI, the marker FJB was utilized in the same tissue. No FJB staining was observed after dSh (Figure 3d4), but an area of FJB-positive neurons was observed in the cortex after dFPI (Figure 3d5).

**Figure 4:**
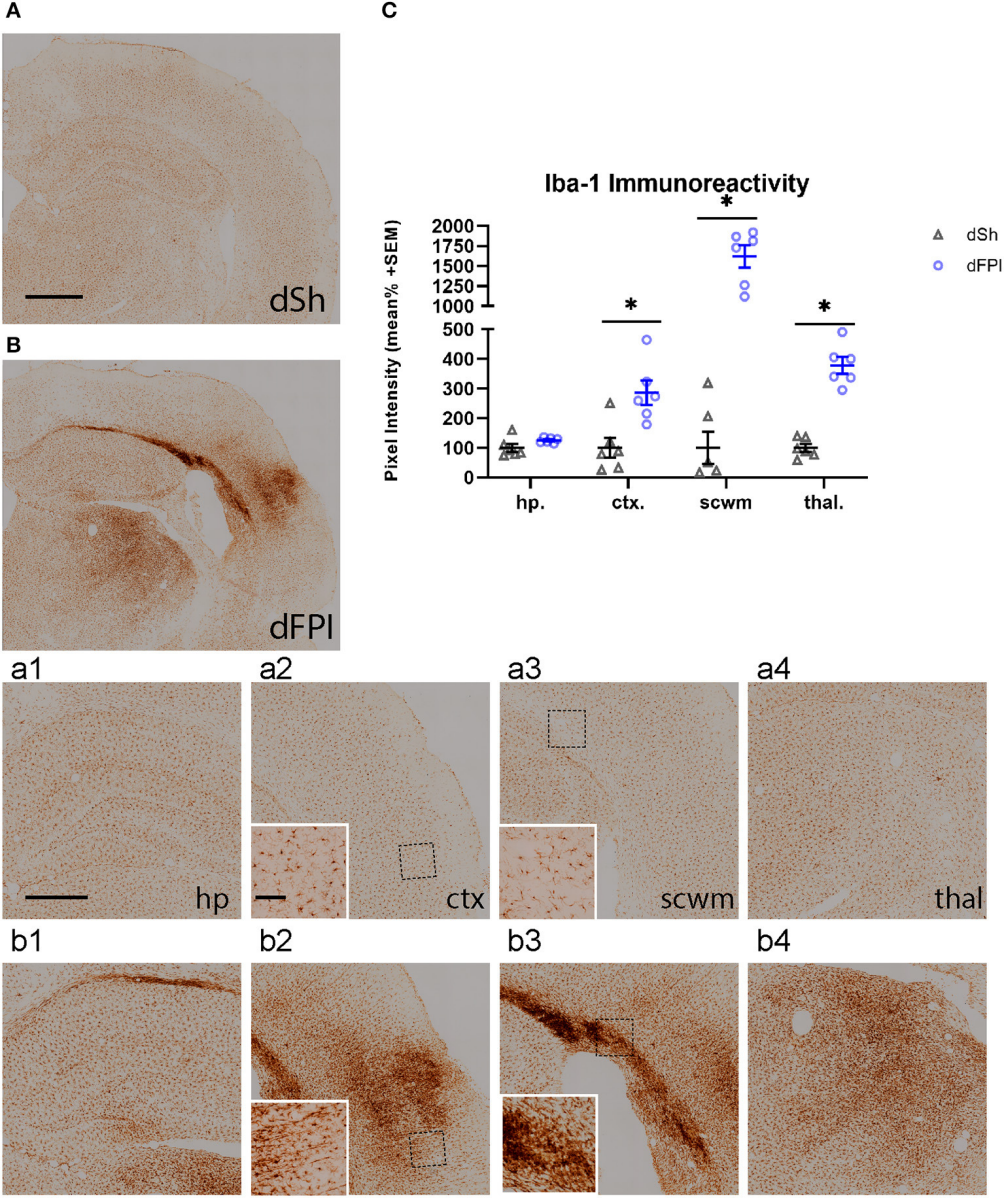
Dual FPI results in increased Iba-1 abundance in the cortex, thalamus, and subcortical white matter 2 weeks post-injury. Representative images of Iba-1 immunoreactivity in the ipsilateral hemisphere at 14 days following dSh **(A)** and dFPI **(B)** are shown (total of *n* = 6 per group), scale bar equal to 1 mm. Representative higher magnification images collected from dSh **(A)** and dFPI **(B)** highlight Iba-1 subregional immunoreactivity in the dSh ipsilateral hippocampus **(a1)**, cortex **(a2)**, subcortical white matter **(a3)**, thalamus **(a4)** and similarly, dFPI subregions **(b1–b4)**. Higher magnification insets are also included to display specific phenomena, such as immunoreactivity in the subcortical white matter (**b2** insert), and the presence of rod microglia in the cortex (**b3** inset). Higher magnification images of **(A,B)** are shown, with scale bars equal to 500 μm **(a1–a4, b1–b4)** and 100 μm (insets shown in **a2,a3,b2,b3**). **(C)** Mean pixel intensity measures revealed a significant increase in the cortex, thalamus, and subcortical white matter following injury (Student's *t*-test, *p < 0.01). Regional analyses were normalized to mean pixel intensity.

**Figure 5:**
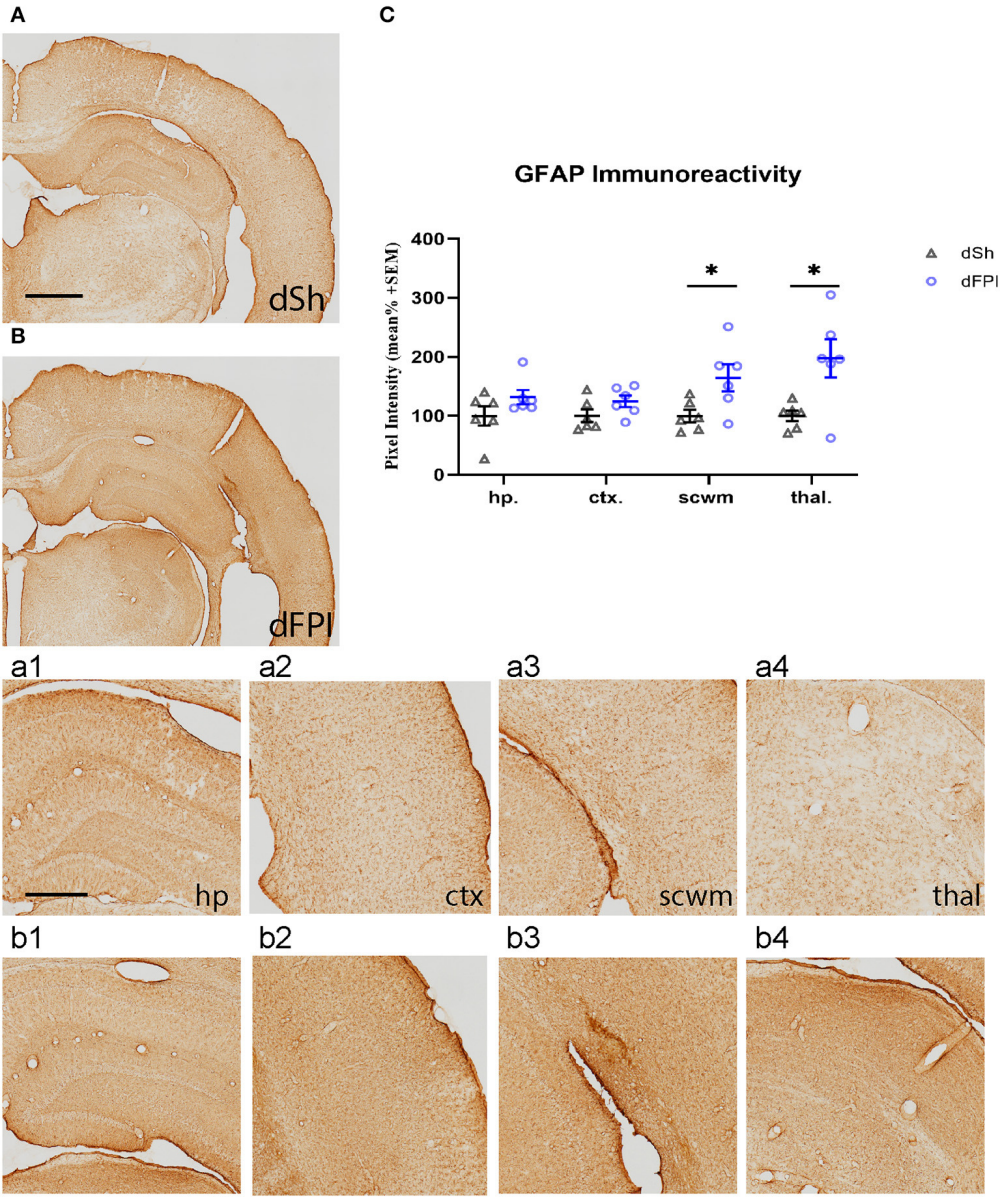
Dual FPI results in increased GFAP abundance in the subcortical white matter and thalamus 2 weeks post-injury. Representative images of GFAP immunoreactivity in the ipsilateral hemisphere at 14 days following dSh **(A)** and dFPI **(B)** are shown (total of *n* = 6 per group), scale bar equal to 1 mm. Representative higher magnification images collected of dSh **(A)** and dFPI **(B)** highlight GFAP subregional immunoreactivity in the dSh ipsilateral hippocampus **(a1)**, cortex **(a2)**, subcortical white matter **(a3)**, thalamus **(a4)** and similarly, dFPI subregions **(b1–b4)**, scale bars equal to 500 μm **(a1–a4,b1–b4)**. **(C)** Mean pixel intensity measures of GFAP immunoreactivity revealed a significant increase in the subcortical white matter and thalamus following dFPI (Student's *t*-test, *p < 0.05). Regional analyses were normalized to mean pixel intensity.

To investigate the neuroinflammatory and glial response at the primary site of injury at 14 days following dFPI, Iba-1 and GFAP immunoreactivity changes were assessed in the hippocampus, cortex, subcortical white matter and thalamus. These regions were defined using the rat brain atlas (44) and analyzed for mean pixel intensity to examine regional changes in microglial immunoreactivity. Representative images of Iba-1 staining for dSh and dFPI are shown (Figures 4A,B). Higher magnification images of subregions (hippocampus, cortex, subcortical white matter, thalamus) collected from dSh (Figures 4a1–a4) and dFPI (Figures 4b1–b4) are shown. Qualitatively, Iba-1 immunoreactivity is increased in multiple regions, including the cortex and thalamus, as well as higher immunoreactivity in the subcortical white matter (Figure 4b3 inset), a region in which hemorrhage is visible in the Cresyl violet stained tissue. Quantitative Iba-1 immunoreactivity measures revealed a significant increase in the cortex and thalamus, with robust increases in the subcortical white matter (Student's *t*-tests; ^*^p < 0.01; Figure 4C). However, measurements of Iba-1 immunoreactivity in the hippocampus revealed no significant changes between sham control and dFPI animals (Figure 4C). Observation of the cortex in dFPI Iba-1 stained images shows the presence of rod-shaped microglia (Figure 4b2 insert), a phenomena previously observed in preclinical models of diffuse TBI (45). Representative images of GFAP staining for dSh and dFPI are shown (Figures 5A,B). Higher magnification images of subregions (hippocampus, cortex, subcortical white matter, thalamus) acquired in dSh (Figures 5a1–a4) and dFPI (Figures 5b1–b4) are shown. Qualitatively, GFAP immunoreactivity appears to be elevated in areas in proximity to Iba-1 changes. Quantitative GFAP immunoreactivity measures revealed a significant increase in the subcortical white matter and thalamus (Student's *t*-tests; ^*^p < 0.05; Figure 5C). Measurements of GFAP immunoreactivity in the hippocampus and cortex revealed no significant changes between dSh and dFPI animals (Figure 5C). To assess for potential increases in inflammatory fluid biomarkers after dFPI and dSh, serum samples collected at termination of the study were tested by ELISA for IL-6 and CCL2. IL-6 levels were undetectable for both dFPI and dSh. Serum levels for CCL2 were detectable at 2 weeks post-injury, but were not significantly different between dFPI (153.3 ± 15.6 pg/ml) and dSh (162.9 ± 11.0 pg/ml).

**Figure 6:**
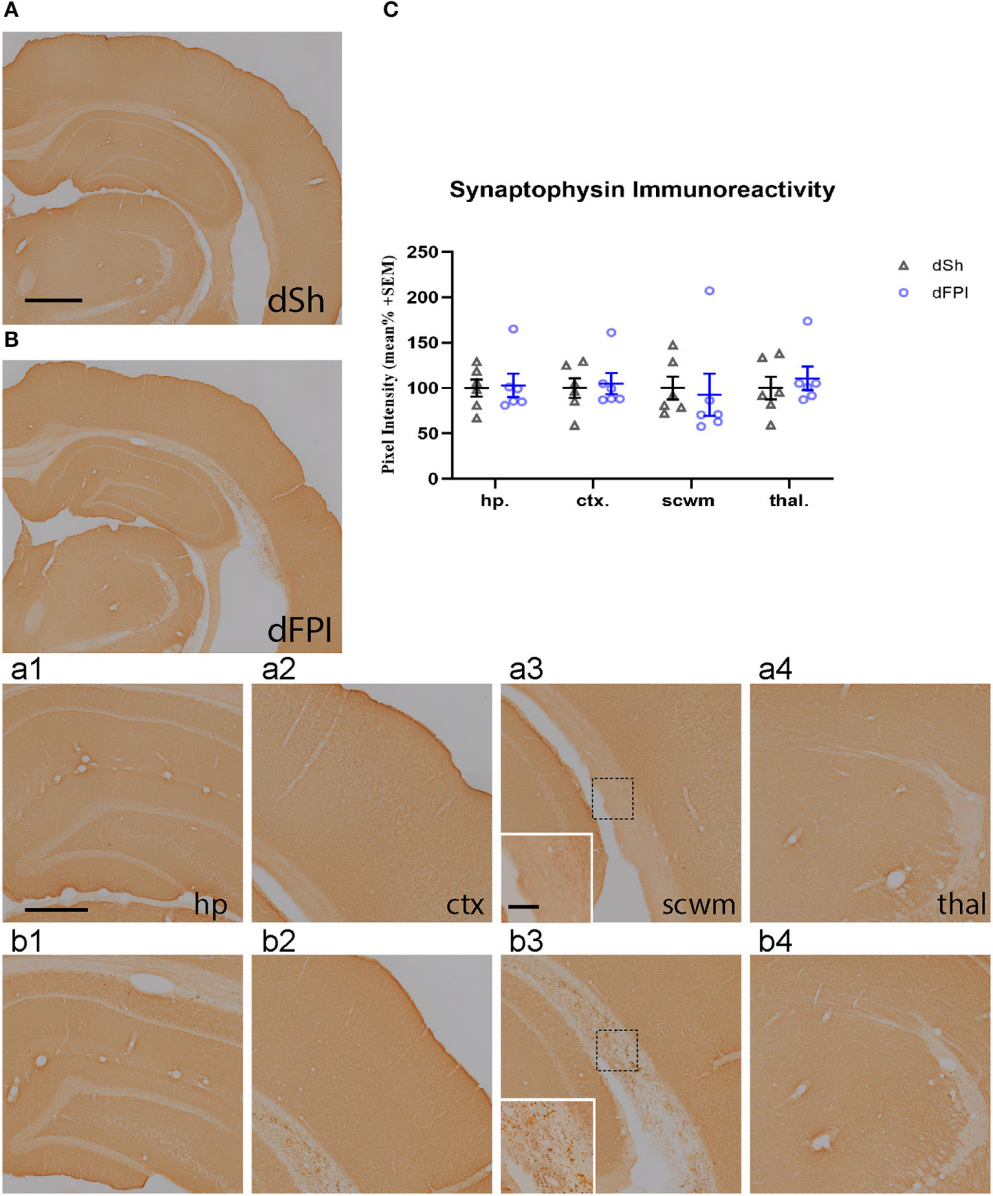
Dual FPI did not result in significant changes in synaptophysin abundance 2 weeks post-injury. Representative images of synaptophysin immunoreactivity in the ipsilateral hemisphere at 14 days following dSh **(A)** and dFPI **(B)** are shown (total of *n* = 6 per group), scale bar equal to 1 mm. Representative higher magnification images collected of of synaptophysin subregional immunoreactivity in **(A,B)** show immunoreactivity in the dSh ipsilateral hippocampus **(a1)**, cortex **(a2)**, subcortical white matter **(a3)**, thalamus **(a4)**, and similarly, dFPI subregions **(b1–b4)**. Higher magnification insets of **(a3,b3)** are shown to display punctate staining in the subcortical white matter, scale bars equal to 500 μm **(a1–a4,b1–b4)** and 100 μm (insets shown in **a3,b3**). **(C)** Mean pixel intensity measures revealed no significant changes in immunoreactivity in any regions of sham and dFPI-injured rats. Regional analyses were normalized to mean pixel intensity.

**Figure 7:**
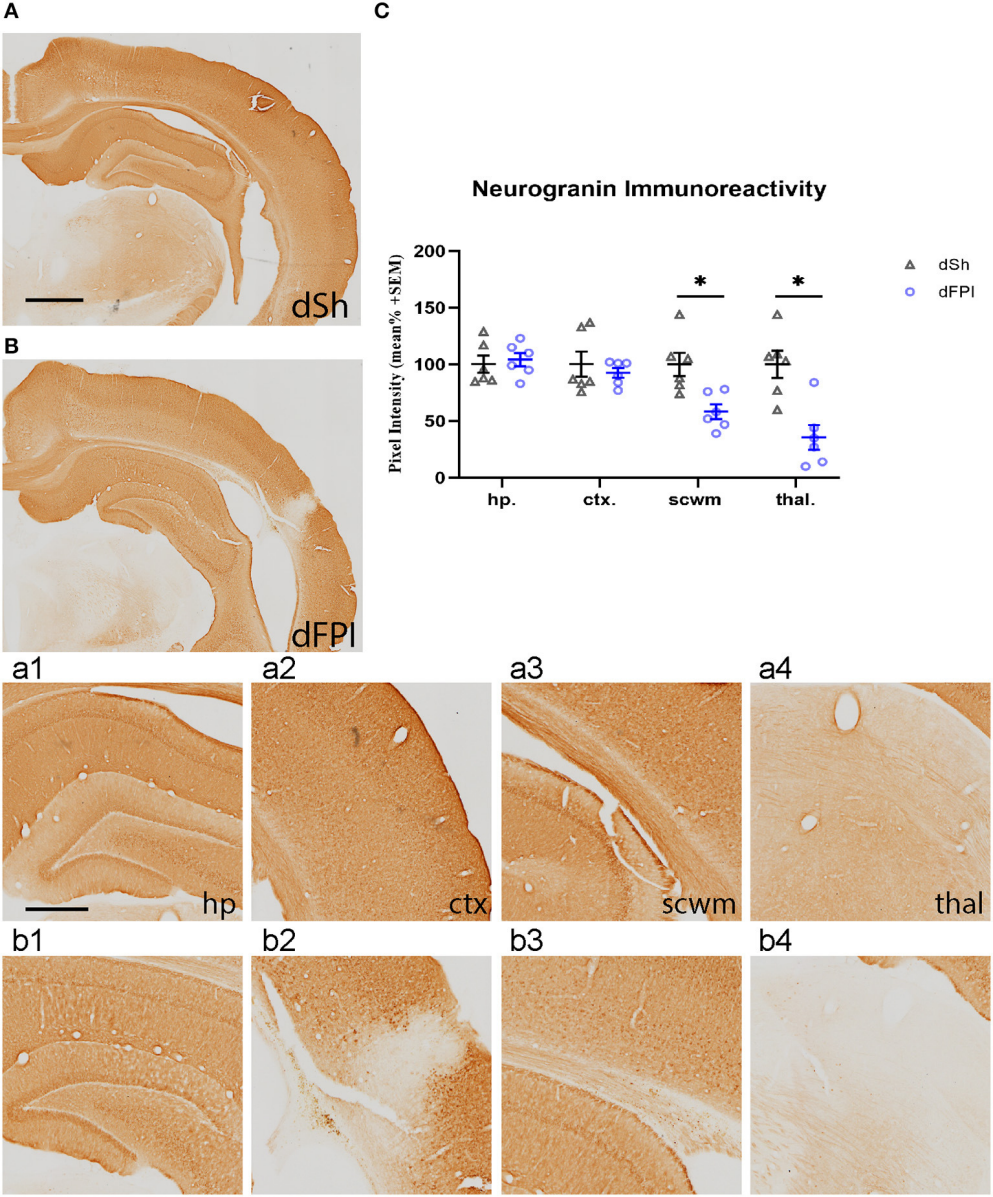
Dual FPI resulted in significant reductions in neurogranin abundance 2 weeks post-injury. Representative images of neurogranin immunoreactivity in the ipsilateral hemisphere at 14 days following dSh **(A)** and dFPI **(B)** are shown (total of *n* = 6 per group), scale bar equal to 1 mm. Representative higher magnification images collected of neurogranin immunoreactivity in **(A,B)** show immunoreactivity in the dSh ipsilateral hippocampus **(a1)**, cortex **(a2)**, subcortical white matter **(a3)**, thalamus **(a4)**, and similarly, dFPI subregions **(b1–b4)**, scale bar equal to 500 μm. **(C)** Mean pixel intensity measures revealed significant reductions in neurogranin immunoreactivity in the subcortical white matter and thalamus of dFPI-injured rats, as compared to dSh rats (Student's *t*-test, *p < 0.05). Regional analyses were normalized to mean pixel intensity.

To investigate changes in synaptic protein abundance in response to dFPI, immunoreactivity of synaptophysin (presynaptic protein) and neurogranin (postsynaptic protein) were assessed in the hippocampus, cortex, subcortical white matter and thalamus. These regions were defined using the rat brain atlas (44) and analyzed for mean pixel intensity to examine regional changes in synaptophysin and neurogranin. Representative images of synaptophysin staining for dSh and dFPI are shown (Figures 6A,B). Higher magnification images of subregions (hippocampus, cortex, subcortical white matter, thalamus) for dSh (Figures 6a1–a4) and dFPI (Figures 6b1–b4) are shown. Measurements of synaptophysin immunoreactivity revealed no significant changes in synaptophysin abundance between sham and injured animals for all regions assessed (Figure 6C). However, qualitative assessment revealed punctate immunoreactivity in the subcortical white matter in all animals after dFPI (6 out of 6), suggestive of a change in synaptophysin localization, possibly an indication of impaired trafficking of synaptophysin in axons (Figure 6b3 inset). Representative images of neurogranin immunoreactivity for dSh and dFPI are shown (Figures 7A,B). Higher magnification images of subregions (hippocampus, cortex, subcortical white matter, thalamus) of dSh (Figures 7a1–a4) and dFPI (Figures 7b1–b4) are shown. Qualitative observations show that despite no significant reduction in cortical neurogranin immunoreactivity was measured after dFPI, there appeared to be an area of pronounced loss near the site of primary injury (6 out of 6; Figure 7b2), as well as an apparent increase in punctate immunoreactivity in subcortical white matter in all animals following dFPI (6 out of 6; Figure 7b3), possibly an indication of impaired trafficking similar to the pattern observed with synaptophysin. Quantitative assessment of neurogranin immunoreactivity revealed no significant changes in hippocampal abundance between sham and dFPI animals (Figure 7C); however, a significant reduction in neurogranin abundance was observed in the subcortical white matter and the thalamus in dFPI animals (Student's *t*-tests; ^*^p < 0.05; Figure 7C).

## Discussion

With the current study, we described the sub-acute behavioral response of rats receiving one, two, or four mild fluid percussion injuries or sham control surgeries. Motor performance showed that the degree of behavioral dysfunction worsened with each additive injury, demonstrating what has been described as the compounding effect of repeat mild injury. Assessment of spatial learning performance revealed significant impairment following the qFPI compared to shams, and a modest, but not significant, trend in the dFPI group. Qualitative assessment of Cresyl violet staining showed that overt tissue damage occurred after qFPI, and limited overt tissue damage occurred following sFPI or dFPI. Based on these behavioral, post-injury weights, and histopathological observations, we described synaptic density changes and neuroinflammatory responses following dFPI compared to dSh, as it reflects a clinically relevant representation of rmTBI. Immunoreactivity measures showed significant reductions in postsynaptic abundance of neurogranin in the subcortical white matter and thalamus in dFPI rats compared to dSh rats. Striking increases in microglia immunoreactivity were observed in the cortex, thalamus, and subcortical white matter following injury, and increased astrocytic immunoreactivity in the subcortical white matter and thalamus after dFPI. Our findings document the behavioral and physiological dysfunction occurring in response to two mTBIs induced by fluid percussion injury and highlight the ability of this rmFPI model to reproduce clinically relevant pathological outcomes.

The scaled motor performance among the different injury groups demonstrates the cumulative effect of injury in this rmFPI model. These outcomes are consistent with other closed and open skull models of mTBI and rmTBI, including other lateral FPI, midline FPI, Marmarou, CHIMERA, and other models. Measures of weight, apnea time, and righting time are also consistent with reports of closed and open skull mTBI studies (35, 38). Most studies of single mTBI report mild or non-significant behavioral dysfunction, while studies of repeated mTBI tend to show behavioral impairment (18–22, 24, 25, 46, 47). Studies investigating rmTBI similarly describe compounding severity of dysfunction with multiple injuries (18–21, 24, 46). A source of variability in reports of motor dysfunction may be rooted in inter-injury interval differences, with longer periods between impacts showing less severe or non-existent behavioral abnormalities (18–22, 24, 25, 27, 46, 48, 49). Our observation of behavioral impairment highlights the relevance of this model in the mTBI literature, as the outcomes align with other reports, along with defining outcomes specific to these injury parameters.

Qualitative analysis of Cresyl violet staining revealed evidence of hemorrhaging and regions of tissue loss at the interface between the gray and white matter following four mild FPI. However, while the incidence of hemorrhage in the dFPI group was similar to qFPI, the prevalence of hemorrhage within the white matter and magnitude of damage appeared to be reduced after fewer injuries, as further suggested by a reduction in incidence in the sFPI animals. These outcomes are compatible with other preclinical studies of rmTBI, as it is common for additional injuries to produce histological damage atypical of a single mild injury (3, 26, 38, 50–53). However, in terms of clinical relevance, it is understood that mild brain injury is characterized in part by lack of lesion formation, visible by imaging, at the time of behavioral or physiological impairment (2, 9), which have been corroborated by experimental models of mTBI (20, 22, 24, 29, 32, 34, 54–59). With this in mind, we chose to further investigate the histological outcomes of animals in cohort 2, those receiving dFPI or dSh control surgeries. In this regard, dFPI represents a clinically aligned model for many reasons, including its societal relevance, as receiving two concussions in short succession is more probable than receiving four (60), and its reduced relative tissue damage, reduced apnea times and limited weight loss. While we did observe FJB positive neurons in the injured cortex after dFPI, this was not accompanied with the formation of an overt lesion. However, we cannot understate the relevance of a higher frequency mFPI model, such as the qFPI. Though it is not directly aligned with the goal of this study, it could be useful in other applications of rmTBI models, where it might be advantageous to produce accelerated histological outcomes, such as in investigations of therapeutic interventions or testing of mechanistic underpinnings of repeated injury.

With regard to dFPI pathophysiology, we observed neuroinflammatory changes in response to dFPI via Iba-1 and GFAP immunoreactivity. We have shown that two mild lateral fluid percussion injuries lead to significant microglial responses in the ipsilateral cortex, as well as increases in Iba-1 and GFAP immunoreactivity in the ipsilateral subcortical white matter and thalamus. Our findings suggest that the degree of neuroinflammation caused by two mild FPI corroborates reports for single mild and rmTBI in rodents. Closed and open skull models of mTBI and rmTBI show varying degrees of neuroinflammation, with most reporting intense microglial and astrocytic activation sub-acutely in the corpus collosum, entorhinal cortex, cerebral cortex, and thalamus (24, 29, 34, 46, 55, 56). Several reports describe similar striking increases, with Witkowski and colleagues (58) showing peak Iba-1 upregulation, immediately following a second mild injury occurring 24 h after the first injury in a mouse Marmarou model of TBI, corroborating our utilization of this dual impact model. Additionally, Mouzon and colleagues (32) showed robust focal microglial activation in the cortex and corpus callosum at a similar timepoint following five mTBI in mice. A few studies utilizing single mild midline or lateral fluid percussion injury similarly recounted extensive microgliosis in white matter and thalamus in the absence of cell loss (22, 57, 59). It has been widely understood that post-TBI neuroinflammation, and the increased abundance of activated microglia, can lead to degenerative events including blood brain barrier disruption, edema, progressive vascular injury, neutrophil respiratory burst, B-cell activation, necrosis, apoptosis, and the production of reactive oxygen species (16, 61–64). Microgliosis has previously been associated with diffuse axonal injury (DAI), with rodent studies of mTBI demonstrating parallel microglial activation and axonal transport disruption in thalamocortical relays, and with an investigation of micro pigs reporting physical convergence of proximal axonal swellings undergoing DAI and activated microglial processes in the thalamus, in the acute phases of injury response (65). This relationship may account for the almost 18 fold increase in Iba-1 immunoreactivity in the subcortical white matter seen in the present study. Thomas and colleagues (57) investigated neuroinflammatory outcomes at a later time point of 28 d post-injury, showing persistent microgliosis past acute stages of injury, suggesting that activated microglia may support the maladaptive circuit reorganization following diffuse TBI, as demonstrated in the thalamic whicker barrel circuit. Further investigations of the dFPI model will seek to investigate the relationship between microgliosis and axonal injury following injury. In terms of repeated brain injury specifically, Huang and colleagues' investigation of post-concussive vulnerability demonstrated that microglial activation exhibits compounding severity in response to repeated head injury when two injuries occur within 3 days or less of each other; however this effect diminishes with two injures occur over a period longer than 3 days (20). This illustrates a window of vulnerability, which is a cornerstone of compounding pathology following rmTBI.

Assessment of the postsynaptic protein neurogranin exhibited significant reductions in the subcortical white matter and thalamus, and an observable reduction in the cortex at the primary site of injury. Alternatively, synaptophysin immunoreactivity, measured in the cortex, hippocampus, thalamus, and subcortical white matter, showed limited changes following dual injury compared to sham animals. Synaptophysin is frequently utilized as a molecular marker for the synapse and a relative indicator of synaptic density changes (66–69). Synaptophysin analysis and Cresyl violet staining of dFPI brains suggest no overt tissue damage or synapse loss as a product of the injury. Injury models exhibiting greater magnitudes of tissue damage show reductions in synaptophysin following TBI (66, 70, 71). Seeing that a behavioral phenotype was produced in our dFPI rats, in absence of reduced synaptic density and visible tissue damage, the pathology may manifest in the form of aberrant synaptic functioning rather than tissue loss. Neurogranin is involved in fine-tuning synaptic plasticity and altered protein expression results in impaired learning and memory outcomes (72). We recently showed that the abundance of neurogranin is reduced in the hippocampus for weeks following TBI in the controlled cortical impact model (73). Distinct expression patterns between synaptophysin and neurogranin was supported by other studies of single mTBI showing abnormal synaptic organization and functioning in the absence of cell loss and synaptic density changes, with a CHIMERA model showing dense presynaptic material and degenerating synaptic boutons following injury (74). On a similar note, high frequency models of mTBI (30 impacts) have shown a decrease in neuron excitability and synaptic plasticity in parallel with cognitive impairment (75). In the current study, though synaptophysin abundance remains the same in multiple regions, there is robust punctate staining in the subcortical white matter of dFPI animals, not present in sham animals. This may be an indicator of altered axonal transport, as synaptophysin has been used similarly to APP as a marker of such phenomena (76). Synaptic dysfunction may be a likely contributor to abnormal neurological functioning following rmTBI and it will be further investigated in future studies. Interestingly, cortical rod shaped microglia were observed in an adjacent region to the synaptophysin punctate staining in the subcortical white matter (Figure 4b2). Rod shaped microglia represent a form of activated microglia and have been known to align with adjacent neurons following diffuse TBI, possibly playing a role in ‘synaptic stripping’, a process of removing damaged synaptic inputs (45, 77, 78). Future studies will further investigate the potential contribution of microglial interactions on synaptic and axonal integrity in this model.

Preclinical studies of mTBI and rmTBI suggest that the concussed brain experiences a period of vulnerability following a single mild injury, wherein a second mild insult occurring within a specific time interval can produce behavioral impairment and pathological outcomes (i.e., cellular disturbances and histological damage) typical of a more severe TBI (26, 38, 48, 50–53, 79). In preclinical models of rmTBI, lengthening the inter-injury interval from 1 day to 2 or 3 days can result in a smaller cortical lesion, dampened microglial and astrocytic activation, and decreased vascular dysfunction, with a 7 day inter-injury interval producing outcomes similar to a single mTBI. This identifies a specific window of vulnerability where additional insults occurring within a time period will lead to compounding pathology, while those occurring outside the window can act as independent events (8, 20, 50, 80). This supports the tissue damage we observed following the qFPI, as it is common for additional injures occurring within the period of vulnerability to produce histological damage atypical of a single mild injury (26, 38, 50–53, 79). Similarly, it explains the compounding severity of behavioral dysfunction following each additional injury observed in our investigation and the variability in behavioral outcomes observed across studies.

In several of these reports mentioned above, this period of vulnerability has been characterized in terms of metabolic perturbation. Vagnozzi and colleagues demonstrated that two mTBI exacerbated disturbances of cellular oxidative metabolism in the rat brain, to a degree similar to a severe TBI. They found that cellular metabolic dysfunction was only exacerbated by a second injury when the additional insult occurred at 3 days, with a second injury at 7 days producing impairment comparable to a single mTBI (38). Prins and colleagues showed that adolescent rats receiving two mTBI 24 h apart exhibited a sustained increase in post-concussive glucose hypometabolism, while insults occurring at an interval of 5 days produced metabolic depression typical of a single concussion, similarly identifying a period of metabolic vulnerability following mild injury and suggesting that a second mTBI introduced after the period of metabolic recovery will act independently of the first, without compounding disturbances (35). The duration of glucose hypometabolism following TBI has also been shown to increase with age, suggesting that the timeline for post-concussive metabolic vulnerability and recovery may be age dependent (30, 81, 82). It has been suggested that this disrupted cellular metabolism following mTBI may render the brain vulnerable to additional traumatic events (53). This disruption may very well be a primary cause of the graded histopathology and behavioral dysfunction observed with additive injuries in the current study.

We chose to implement the lateral FPI model to highlight its utility and reliability in producing clinically translatable repeated mTBI. Closed head injury models have been largely employed compared to open head models in studies mTBI and rmTBI; they have been favored due to their ability to produce diffuse mild injury without jeopardizing the integrity of the skull, as is reflective of human mTBI. Ondek and colleagues recently developed a rat model of diffuse mTBI utilizing a metal disc secured to the intact closed skull, and delivering an impact using a CCI device; they show that this model affords the precise control and replicability of CCI, while also avoiding skull fracture sometimes observed with traditional closed head models (8). Other models focus on producing rotational acceleration prevalent in clinical concussion, with animal models being produced specifically to replicate this phenomena, such as the CHIMERA model (24, 74). While FPI does require disrupting the cranial vault, the closed head environment can be imitated during injury with the skull being sealed by the injury device (17). The open skull then also allows for highly specific injury localization, making it a successful and reliable model for replicating clinical features of closed head injuries. The FPI model is flexible in that localization can be shifted precisely to induce differing injury profiles. Typically FPI models are used in studies of mTBI, where midline FPI is often utilized over a lateral FPI, due to its ability to produce diffuse injury, the absence of a lesion, and prevalent axonal injury, which is advantageous for studies investigating TBI-induced DAI. Lateral impact affords the ability to shift the injury over the ipsilateral hippocampus, supporting an investigation of hippocampal dependent learning and memory outcomes as a result of rmTBI. For these reasons, the lateral FPI model can provide utility in studying features of rmTBI.

Taken together, the literature suggests that variability between rmTBI models may be attributed to differences in injury parameters, such as frequency and inter-injury interval, and perhaps less due to the employment of a closed vs. and open head model. As models are implemented to align with the needs of the particular research question, each model has certain advantages that may be relevant for varying studies. We have described the dual FPI in this study to show pathological outcomes at the onset of cumulative vulnerability, and also provide insight into an additional rat rmTBI model for future investigations.

In summary, this study has provided a novel account of the sub-acute post injury outcomes occurring in response to a rmFPI with these parameters. Assessment of behavioral dysfunction revealed a significant difference in motor performance between groups receiving sFPI, dFPI, and qFPI, demonstrating the cumulative effect of repeat mild injury. Assessment of Cresyl Violet staining showed that overt tissue damage occurred following qFPI compared to shams, and reduced damage occurred following sFPI or dFPI. Neurogranin immunoreactivity exhibited subregional reductions, while synaptophysin immunoreactivity measures showed no significant changes in synaptic density in dFPI rats compared to dSh rats, but both proteins showed possible signs of disrupted axonal trafficking in the subcortical white matter. Iba-1 showed striking increases in microglia immunoreactivity in the cortex, while both Iba-1 and GFAP showed increased subcortical white matter and thalamic immunoreactivity following dFPI. These findings demonstrate the behavioral and physiological dysfunction occurring in response to dual mTBI induced by fluid percussion injury and show the reliability of the lateral FPI in producing clinically relevant rmTBI.

## Data availability statement

The raw data supporting the conclusions of this article will be made available by the authors, without undue reservation.

## Ethics statement

The animal study was reviewed and approved by University of Pittsburgh Institutional Animal Care and Use Committee.

## Author contributions

KF, AR, SS, MP, EH, JH, CD, and SC completed the investigation and reviewed the manuscript. KF and SC completed data curation and analysis. KF, SS, CD, and SC drafted, edited, and revised the manuscript. All authors contributed to the article and approved the submitted version.

## Funding

This work was supported by the National Institutes of Health Grant 1R21NS111099 (SC), 1R21NS115440 (CD), The Chuck Noll Foundation 008 (SC) and 002 (CD), the Veterans Health Administration I01 BX005291 (CD), and the Walter L. Copeland Fund of The Pittsburgh Foundation (SC).

## Conflict of interest

The authors declare that the research was conducted in the absence of any commercial or financial relationships that could be construed as a potential conflict of interest.

## Publisher's note

All claims expressed in this article are solely those of the authors and do not necessarily represent those of their affiliated organizations, or those of the publisher, the editors and the reviewers. Any product that may be evaluated in this article, or claim that may be made by its manufacturer, is not guaranteed or endorsed by the publisher.
